# Intra- and extra-work psychosocial factors in physicians performing mandatory social service in Nariño, Colombia

**DOI:** 10.47626/1679-4435-2025-1429

**Published:** 2025-08-25

**Authors:** Angie Ximena Ortiz-Chamorro

**Affiliations:** 1 School of Medicine, Universidad CES, Medellín, Antioquia, Colombia

**Keywords:** psychosocial impact, occupational health, social work, physicians, COVID-19, impacto psicosocial, salud laboral, servicio social, médicos, COVID-19

## Abstract

**Introduction:**

Psychosocial risk among mandatory social service physicians is a relevant concern due to work demands and adverse conditions.

**Objectives:**

To identify intra- and extra-work psychosocial factors affecting physicians who completed their mandatory social service in different regions of the department of Nariño, Colombia, during the COVID-19 pandemic.

**Methods:**

This was an observational, descriptive, cross-sectional study based on primary data sources. A total of 104 mandatory social service physicians from Nariño, southwestern Colombia, were surveyed using the Intra- and Extra-Work Psychosocial Risk Questionnaires from the psychosocial risk batery developed by the Ministry of Health and Social Protection of Colombia. Absolute and relative frequencies were calculated for each variable of interest. Total risk scores, as well as intra- and extra-work risk dimensions, were analyzed and compared with previous studies.

**Results:**

Overall, 79.8% of physicians were classified as being at very high psychosocial risk, while 78.8% were at very high risk for intra-work factors, with the most affected dimensions being “leadership,” “job demands,” and “rewards”. Additionally, 39.4% were at very high risk for extra-work factors, with “influence of the extra-work environment on work” being the most affected dimension. The “family relationships” dimension showed a low risk in 49% of participants.

**Conclusions:**

The high prevalence of psychosocial risk, especially in emotional demands and leadership, highlights the urgent need for implementation of intervention strategies within an epidemiological surveillance system. It is recommended to prioritize actions aimed at mitigating the impact of stress on these professionals, considering their essential role in health care.

## Abstract (pt)

**Introducción:**

El riesgo psicosocial en médicos de servicio social es un problema relevante debido a las exigencias laborales y condiciones adversas.

**Objetivos:**

Identificar los factores psicosociales intralaborales y extralaborales en médicos que realizaron su servicio social obligatorio en diferentes regiones del departamento de Nariño durante la pandemia por COVID-19.

**Métodos:**

Se trata de un estudio observacional, descriptivo, de corte transversal, de fuente primaria de información. Se incluyeron a 104 médicos en servicio social en el departamento de Nariño, suroccidente colombiano, a quienes se les aplicaron los cuestionarios de riesgo psicosocial intralaboral y extralaboral de la batería de riesgo psicosocial del Ministerio de Salud y Protección Social de Colombia. Se calcularon frecuencias absolutas y relativas para cada variable de interés. Se analizaron los puntajes totales y las dimensiones de riesgo intralaboral y extralaboral, comparándolos con estudios previos.

**Resultados:**

El 79,8% de los médicos presentó un nivel de riesgo psicosocial muy alto y el 78,8% se clasificó en riesgo muy alto para factores intralaborales, con mayor afectación en las dimensiones de liderazgo, demandas laborales y recompensas. En el ámbito extralaboral, el 39,4% presentó riesgo muy alto, siendo influencia del entorno extralaboral en el trabajo la dimensión más afectada. La dimensión relaciones familiares mostró un riesgo bajo en el 49% de los participantes.

**Conclusiones:**

La alta prevalencia de riesgo psicosocial, especialmente en demandas emocionales y liderazgo, indica la necesidad de implementar estrategias de intervención en un sistema de vigilancia epidemiológica. Se recomienda priorizar acciones para mitigar el impacto del estrés en estos profesionales, considerando su rol esencial en la atención sanitaria.

## INTRODUCTION

Psychosocial factors are present in all types of organizations and derive from the general conditions of their management practices. Such factors can be either favorable or unfavorable, depending on whether they benefit work organization and support employees’ professional development or pose risks to the health and/or well-being of workers (in this case, they are referred to as psychosocial risk factors).^1^

The International Labour Organization defines psychosocial factors as the interactions between the work environment, job content, and organizational conditions, among others. Therefore, psychosocial risk refers to different factors that affect the professional and personal dimensions of workers’ lives, including performance and motivation.^2^

According to Article 5 of Resolution 2646 of 2008, issued by the Ministry of Health and Social Protection of Colombia, psychosocial factors comprise intra-work, extra-work, and individual aspects. These factors are interrelated and can affect health and performance in the work seting.^3^

Practicing medicine can bring great satisfaction when working conditions are favorable and appropriate. However, work environments that expose physicians to psychosocial risk factors can lead to dissatisfaction, high stress levels, time pressure, emotional exhaustion, and physical and mental health problems.^4^

Physicians are exposed to several psychosocial risk factors at work, as they often face not only work overload but also a lack of personnel and work equipment.^5^ In turn, primary health care physicians working in rural health care centers must cope with the constant increase in the number of re-consultations.^6^ Therefore, psychosocial factors that emerge from the interaction between internal and external work elements, as well as from individual aspects, can affect cognition (e.g., reduced concentration and memory), mood (e.g., depression, anxiety, or distress), behavior (e.g., use of psychoactive substances), and physiology (e.g., physical and psychological disorders).^7^

Among physicians, the most common psychosocial risk factors are perceived lack of control, lack of support, low reward, and high demands. Furthermore, primary health care physicians receive less organizational support than hospital-based physicians, who, conversely, face greater demands.^8^

In Colombia, social service is a mandatory requirement for medical practice. It also serves as a mechanism for newly graduated physicians from private and public institutions to apply the knowledge obtained during undergraduate studies and to have their first experience in the labor market.^9^ Through this new experience, physicians begin their professional practice and are exposed to different psychosocial risk factors at work due to overload, limitations of the Colombian health system, and shortages of personnel to meet patient demand.^8^

Therefore, the aim of this study was to identify intra- and extra-work psychosocial factors among physicians who completed their mandatory social service in different regions of the department of Nariño, southwestern Colombia, during the COVID-19 pandemic, using a batery of psychosocial risk tests.

## METHODS

### DESIGN, TYPE OF STUDY, AND POPULATION

This was an observational, descriptive, cross-sectional study that assessed intra- and extra-work psychosocial factors among physicians from Nariño, Colombia. Potential participants included 147 physicians who were registered to perform social service by the Ministry of Health and Social Protection of Colombia in the department of Nariño from 2021 to 2022. A total of 104 physicians (population census) who met the inclusion criteria were interviewed.

### DATA COLLECTION INSTRUMENTS

Structured interviews were conducted using the following instruments: Intra-Work Psychosocial Risk Questionnaire – Form A and Extra-Work Psychosocial Risk Questionnaire, both of which are included in the battery of psychosocial risk tests published in 2010 and developed and validated by the Ministry of Health and Social Protection of Colombia and the Social Security and Professional Risk Subcenter of Pontificia Universidad Javeriana^10^ for use with workers in Colombia. Both questionnaires were validated in the country with workers affiliated with the General System of Occupational Risks (*Sistema General de Riesgos Profesionales*).

The intra-work questionnaire was designed to evaluate work-related conditions, work organization, and the work environment, which may have no positive effects on workers’ health and professional development when presenting certain characteristics.^10^ This questionnaire is composed of four domains, which in turn are composed of 19 dimensions that represent sources of psychosocial risk at work. Items from the questionnaire are rated on a Likert scale with the following answer choices: always, almost always, sometimes, almost never, and never, with scores ranging from 0 to 4.

The extra-work questionnaire was designed to assess conditions external to the work environment, which are related to workers’ family, social, and economic environment, through seven dimensions.

The rating and interpretation of items from both questionnaires were performed according to the user’s manual for the Intra-Work Psychosocial Risk Questionnaire.^10^ For this purpose, an external professional was involved (a psychologist with a master’s degree in occupational safety and health and a valid license to provide services in occupational psychology), following the batery’s recommendations and in compliance with Resolution 2646 of 2008.^11^

The Intra-Work Psychosocial Risk Questionnaire – Form A, applicable to workers in professional positions, consisted of 123 items, whereas the Extra-Work Psychosocial Risk Questionnaire consisted of 31 items.

### DATA COLLECTION

The instruments were heteroadministered to social service physicians participating in the study (the investigator was the only interviewer) after informed consent was obtained in person when logistic and pandemic conditions allowed. Otherwise (i.e., in most cases), the interview was conducted online through Google Meet video calls, with the support of Google Forms. The investigator was previously trained in reading the batery of tests, as well as the recommendations and guidelines for its administration. Moreover, in compliance with Resolution 2646 of 2008, the results obtained in the instruments were reviewed by a psychologist with a master’s degree in occupational safety and health, who was not a member of the research team and acted as an external evaluator. Data were collected from October 2021 to March 2022.

### VARIABLES

Sociodemographic variables included age, sex, marital status, place of residence, and household composition.

The following dimensions were considered as target variables for intra-work psychosocial risks: a) Job demands – environmental and physical effort demands, emotional demands, quantitative demands, influence of the job on the extra-work environment, job responsibility demands, mental workload demands, role consistency, and workday demands; b) Control over work – role clarity, training, participation and change management, opportunities for the development and use of skills and knowledge, and control and autonomy over work; c) Leadership and social relationships at work – leadership characteristics, social relationships at work, performance feedback, and relationship with colleagues; and d) Rewards – rewards derived from belonging to the organization and the work performed, recognition, and compensation.

The Extra-Work Psychosocial Risk Questionnaire assesses the following dimensions: commuting from home to work and vice versa, characteristics of the household and its surroundings, time away from work, communication and interpersonal relationships, family relationships, influence of the extra-work environment on work, and family economic situation.

### DATA ANALYSIS

Sociodemographic, intra-work, and extra-work variables were expressed as absolute and relative frequencies. Summary variables were calculated using measures of position (median, quartiles, and percentiles), after applying the Kolmogorov-Smirnov test to check for normality of data for the quantitative variable age. Analyses were performed using Jamovi, version 2.3, a free statistical package.

### ETHICAL CONSIDERATIONS

This research was approved by the Institutional Human Research Ethics Commitee of Universidad CES, according to Opinion No. 169 of September 30, 2021. The study adhered to the ethical principles set forth in the ethical regulation on human research in Colombia (Resolution 008430 of 1994) and complied with the principles set forth in the Declaration of Helsinki. Informed consent was obtained from all participants, and they received an email with recommendations based on their scores and results obtained in the risk assessment batery.

## RESULTS

Of the 104 mandatory social service physicians, 65.4% were women and 34.6% were men. Furthermore, 90.4% were single, 52.9% lived in the urban area at the time of social service, and 56.7% lived with one or more people. Finally, 50% were aged 26 years or younger, with a minimum age of 22 years and a maximum age of 40 years (Table 1).

**Table 1:** Sociodemographic characteristics of social service physicians from Nariño, Colombia, 2021-2022

Variables		n	%
Sex			
Female		68	65.4
Male		36	34.6
Marital status			
Single		94	90.4
Married		3	2.9
Separated		1	1.0
Common-law marriage		5	4.8
Widowed		1	1.0
Place of residence			
Rural and semirural		49	47.1
Urban		55	52.9
Household composition			
Lives alone		45	43.3
Lives with others		59	56.7
Variable	Median	Q1	Q3
Age (years)	26	25	28

Q1 = quartile 1; Q3 = quartile 3.

When assessing intra-work psychosocial characteristics by dimensions, it was found that, within the “Leadership and social relationships at work” domain, 59.6% of physicians were classified as having very high risk for the “leadership characteristics” dimension; 25% as having high risk for the “social relationships at work” dimension; and 37.5% as having very high risk for the “performance feedback” dimension (Table 2).

**Table 2:** Description of intra-work psychosocial factors by dimension among mandatory social service physicians from the department of Nariño, Colombia, 2021-2022

Domain	Dimension	Category	Frequency	Percentage (%)
Leadership and social relationships at work	Leadership characteristics	Very high risk	62	59.6
		High risk	24	23.1
		Medium risk	9	8.7
		Low risk	7	6.7
		No risk	2	1.9
	Social relationships at work	Very high risk	19	18.3
		High risk	26	25.0
		Medium risk	26	25.0
		Low risk	14	13.5
		No risk	19	18.3
	Performance feedback	Very high risk	39	37.5
		High risk	20	19.2
		Medium risk	17	16.3
		Low risk	17	16.3
		No risk	11	10.6
	Relationship with collaborators (subordinates)	Very high risk	17	16.3
		High risk	18	17.3
		Medium risk	6	5.8
		Low risk	11	10.6
		No risk	52	50.0
Control over work	Role clarity	Very high risk	43	41.3
		High risk	33	31.7
		Medium risk	9	8.7
		Low risk	10	9.6
		No risk	9	8.7
	Training	Very high risk	31	29.8
		High risk	41	39.4
		Medium risk	11	10.6
		Low risk	6	5.8
		No risk	15	14.4
	Participation and change management	Very high risk	47	45.2
		High risk	28	26.9
		Medium risk	12	11.5
		Low risk	13	12.5
		No risk	4	3.8
	Opportunities for the development and use of skills and knowledge	Very high risk	12	11.5
		High risk	19	18.3
		Medium risk	32	30.8
		Low risk	15	14.4
		No risk	26	25.0
	Control and autonomy over work	Very high risk	86	82.7
		High risk	10	9.6
		Medium risk	4	3.8
		Low risk	4	3.8
		No risk	0	0.0
Job demands	Environmental and physical effort demands	Very high risk	62	59.6
		High risk	25	24.0
		Medium risk	11	10.6
		Low risk	4	3.8
		No risk	2	1.9
	Emotional demands	Very high risk	98	94.2
		High risk	5	4.8
		Medium risk	1	1.0
		Low risk	0	0.0
		No risk	0	0.0
	Quantitative demands	Very high risk	50	48.1
		High risk	36	34.6
		Medium risk	10	9.6
		Low risk	3	2.9
		No risk	5	4.8
	Influence of the job on the extra-work environment	Very high risk	62	59.6
		High risk	15	14.4
		Medium risk	14	13.5
		Low risk	11	10.6
		No risk	2	1.9
	Job responsibility demands	Very high risk	11	10.6
		High risk	24	23.1
		Medium risk	31	29.8
		Low risk	26	25.0
		No risk	12	11.5
	Mental workload demands	Very high risk	25	24.0
		High risk	26	25.0
		Medium risk	18	17.3
		Low risk	21	20.2
		No risk	14	13.5
	Role consistency	Very high risk	53	51.0
		High risk	22	21.2
		Medium risk	12	11.5
		Low risk	12	11.5
		No risk	5	4.8
	Workday demands	Very high risk	85	81.7
		High risk	18	17.3
		Medium risk	0	0.0
		Low risk	1	1.0
		No risk	0	0.0
Rewards	Rewards derived from belonging to the organization and the work performed	Very high risk	57	54.8
		High risk	26	25.0
		Medium risk	8	7.7
		Low risk	7	6.7
		No risk	6	5.8
	Recognition and compensation	Very high risk	62	59.6
		High risk	24	23.1
		Medium risk	8	7.7
		Low risk	8	7.7
		No risk	2	1.9

Within the “Job demands” domain, 59.6, 94.2, 48.1, 59.6, 51, and 81.7% of physicians were classified as having very high risk in the following dimensions: environmental and physical effort demands, emotional demands, quantitative demands, influence of the job on the extra-work environment, role consistency, and workday demands, respectively (Table 2).

Furthermore, 63.5% of physicians were at a very high risk for the “time away from work” dimension; 49% were at a low risk for the “family relationships” dimension; 30.8% were at a high risk for “communication and interpersonal relationships” dimension; 42.3% were at a low risk for the “family economic situation” dimension; 40.4% were a very high risk for the “characteristics of the household and its surroundings” dimension; 38.5% were at a very high risk for the “influence of the extra-work environment on work” dimension; and 36.5% were classified as having no risk for the “commuting from home to work and vice versa” dimension (Table 3).

**Table 3:** Description of extra-work psychosocial factors by dimension among mandatory social service physicians from the department of Nariño, Colombia, 2021-2022

Dimension	Category	Frequency	Percentage (%)
Time away from work	Very high risk	66	63.5
	High risk	22	21.2
	Medium risk	11	10.6
	Low risk	4	3.8
	No risk	1	1.0
Family relationships	Very high risk	1	1.0
	High risk	7	6.7
	Medium risk	0	0.0
	Low risk	51	49.0
	No risk	45	43.3
Communication and interpersonal relationships	Very high risk	30	28.8
	High risk	32	30.8
	Medium risk	14	13.5
	Low risk	17	16.3
	No risk	11	10.6
Family economic situation	Very high risk	18	17.3
	High risk	26	25.0
	Medium risk	0	0.0
	Low risk	44	42.3
	No risk	16	15.4
Characteristics of the household and its surroundings	Very high risk	42	40.4
	High risk	12	11.5
	Medium risk	16	15.4
	Low risk	10	9.6
	No risk	24	23.1
Influence of the extra-work environment on work	Very high risk	40	38.5
	High risk	30	28.8
	Medium risk	7	6.7
	Low risk	14	13.5
	No risk	13	12.5
Commuting from home to work and vice versa	Very high risk	15	14.4
	High risk	14	13.5
	Medium risk	22	21.2
	Low risk	15	14.4
	No risk	38	36.5

An analysis of overall scores for intra-work psychosocial risk factors showed that 78.8% of mandatory social service physicians were classified as having a very high psychosocial risk, and only 1% were classified as having a low risk or no risk. Conversely, an analysis of overall scores for extra-work psychosocial risk factors revealed that 39.4% of physicians were classified as having very high psychosocial risk, and 4.8% were classified as having no risk. When combining the scores for both factors, it was found that 79.8% of physicians had a very high psychosocial risk, and only 1% was classified as having no risk (Table 4).

**Table 4:** Description of intra- and extra-work psychosocial factors among mandatory social service physicians from the department of Nariño, Colombia, 2021-2022, based on overall risk scores

Variable	Category	Frequency	Percentage (%)
Overall score on the intra-work psychosocial risk factors questionnaire	Very high risk	82	78.8
	High risk	15	14.4
	Medium risk	5	4.8
	Low risk	1	1.0
	No risk	1	1.0
Overall score on the extra-work psychosocial risk factors questionnaire	Very high risk	41	39.4
	High risk	25	24.0
	Medium risk	19	18.3
	Low risk	14	13.5
	No risk	5	4.8
Overall score on the intra- and extra-work psychosocial risk factors questionnaires	Very high risk	83	79.8
	High risk	14	13.5
	Medium risk	6	5.8
	Low risk	0	0.0
	No risk	1	1.0

## DISCUSSION

An analysis of overall scores for both intra- and extra-work psychosocial risk (combined) showed that 79.8% of physicians were classified as having a very high psychosocial risk, suggesting a significant psychosocial impact on those completing their mandatory social service in Nariño. This result aligns with the findings of Flórez et al.,^12^ who applied the same psychosocial risk batery to a group of 35 mandatory social service physicians in Antioquia and Caldas, Colombia. The authors found that the total transformed score indicated a very high psychosocial risk in 39.1% of participants. In addition, when analyzing the total scores for the four domains that make up the intra-work psychosocial risk questionnaire, they found a critical level of 43.4%.

Overall, 78.8% of social service physicians in the present study were classified as being at very high risk for intra-work psychosocial factors. This finding is consistent with those by Uribe & Martínez,^13^ who investigated 62 health care professionals from a health care institution in Bucaramanga, Colombia. The authors observed that 76.1% of participants were classified as having a very high psychosocial risk. Additionally, in the “Leadership and social relationships at work” and “Job demands” domains, participants also exhibited very high psychosocial risk, a finding that aligns with the present study, in which the majority of physicians were classified at the same risk level across all four domains of the intra-work questionnaire. Our findings are also consistent with those reported by Morillo et al.,^14^ who investigated psychosocial factors among 143 health care professionals from a hospital in Mocoa, Colombia. In their study, the majority of participants (36%) presented a very high overall psychosocial risk score.^14^

The high risk identified in the “Rewards” and “Job demands” domains (according to their respective dimensions) is consistent with the findings reported by Talavera et al.^8^ in a study of physicians in Valladolid, Spain. Their results showed that all workers were classified as being on alert for the following areas: organizational support, rewards, and demands, with lack of control, lack of support, low rewards, and high demands standing out. However, their study differed from the present study in the instrument used for analysis, which in their study was the DECORE questionnaire.

Results for the “performance feedback,” “role clarity,” and “participation and change management” dimensions reveal that managers do not take physicians’ opinions as employees into account with regard to improving processes. In addition, they fail to inform physicians in a timely manner about changes that could impact their professional practice within the institution where they are completing their mandatory social service.^10^ The “recognition and compensation”, “control and autonomy over work”, “environmental demands and physical effort”, “emotional demands”, “quantitative demands”, “influence of the worker on the extra-work environment”, “role consistency”, “demands of the work day”, “rewards derived from relevance to the organization and the work performed” dimensions, also presented the same risk level (high or very high) in both the present study and the one by Flórez et al.^12^

In the present study, one dimension – “relationship with colleagues” – stood out, as 50% of physicians were classified as having low risk or no risk. This contrasts with the study by Uribe & Martínez,^13^ in which all four dimensions that compose the “Leadership and social relationships at work” domain, including the “relationship with colleagues” dimension, were rated as high psychosocial risk. Our results also differ from those reported by López et al.,^15^ who assessed psychosocial risks among 49 physicians from a clinic in Bogotá, Colombia, during the pandemic. The authors found that 34.5% of participants were at high risk for this dimension.

The present study on mandatory social service physicians also aligns with that of Uribe & Martínez,^13^ as both populations of health care professionals were at very high risk for the “control and autonomy over work” dimension. This suggests that physicians do not have enough autonomy and decision-making power at work in relation, for example, to consultation times and/or patient examination.

Our study is also consistent with several findings reported by Lozano et al.^16^ in a literature review of psychosocial risk factors among health personnel published in 2022. In most of the studies included in the review that administered the batery of psychosocial risk tests, very high risk levels were found in the four domains of intra-work psychosocial risk.

Although most physicians presented very high overall risk scores for both intra- and extra-work factors, the overall extra-work psychosocial risk was below 50%. Specifically, 23.8% of physicians were classified at some level of risk, as follows: 39.4% at very high risk, 24% at high risk, 18.3% at moderate risk, and 13.5% at low risk. Conversely, a study review conducted by Anaya & Polo^17^ found a prevalence of only 10.6% of extra-work psychosocial risks across the studies included in the review.

Moreover, 39.4% of mandatory social service physicians in our sample were classified as having a very high risk for extra-work psychosocial factors, a percentage higher than that found by Ramírez & Barrera,^18^ who reported that 9% of health care professionals were classified as having a very high risk level. In addition, only 4.8% of mandatory social service physicians from our study were classified as having no extra-work psychosocial risk, a percentage significantly lower than the 31% reported by Ramírez & Barrera^18^ among health care professionals from a social company in Boyacá.

The present study found that three out of the seven dimensions of extra-work psychosocial risk presented very high risk scores, in contrast with the study by Pérez et al., in which the only dimension that presented very high risk scores was “time away from work,” affecting 52.7% of participants. Additionally, their study found that 36.4% of participants were at high risk for the “influence of the extra-work environment on work” dimension (unlike the present study, in which this dimension presented a very high risk level).^12^ Furthermore, the present study is similar to the findings of López et al.,^15^ where 31.3% were also classified as high risk for that same dimension; however, for the communication and interpersonal relationships dimension, 30.8% of Nariño physicians were found to be at high risk, higher than the findings of López et al.,^15^ where only 20.7% were at high risk for that dimension.

Conversely, in the present study, the “family relationships” dimension presented a low risk score in 49% of physicians, and only 1% presented very high risk. This suggests that physicians’ interactions with their family are appropriate in most cases. This finding coincides with that observed in the study by López Góngora et al.,^15^ in which most physicians (22.6%) interviewed during the pandemic were also classified as having a low risk for this dimension.

## CONCLUSIONS

This study identified important aspects that reflect the working conditions experienced by mandatory social service physicians in Nariño, promoting important reflections on the mater. High levels of psychosocial risk (as observed in most dimensions in the present study) are highly likely to be associated with a very high response to stress. Hence, these dimensions require relevant interventions within an epidemiological surveillance system, especially in the “emotional demands” dimension, where approximately 9 out of 10 physicians were classified as being at very high risk. Therefore, it is important to consider emotional and affective factors related to physicians’ tasks that may potentially interfere with their emotions and feelings and that may lead to higher risk scores in this dimension. Such factors include patients’ negative emotions or atitudes, contact with injured or dead people, hiding true emotions or feelings at work, or situations such as violence and/or threats.

## References

[1] Bayo BG (2017). La relación existente entre suicidio y profesión ejercida. Especial
referencia al personal sanitario.

[2] Oficina Internacional del Trabajo (OIT) (2019). Factores psicosociales en el trabajo: naturaleza, incidencia y
prevención.

[3] Polanco-Martínez AL, García-Solarte M (2017). Revisión conceptual de los factores de riesgo psicosocial laboral y
algunas herramientas utilizadas para su medición en Colombia. Libre Empres.

[4] Salazar PM (2009). Exposición a factores de riesgo psicosocial, salud, estrés y
satisfacción en médicos residentes y posgradistas que laboran en el
Hospital Eugenio Espejo de la Ciudad de Quito, 2009.

[5] Escribà-Agüir V, Bernabé-Muñoz Y (2002). Exigencias laborales psicológicas percibidas por médicos
especialistas hospitalarios. Gac Sanit.

[6] Sandín-Vázquez M, Conde-Espejo P (2011). Hiperfrecuentación: percepción de los profesionales de
atención primaria sobre la influencia de factores sociales y de
organización del entorno sanitario. Rev Calid Asist.

[7] Rueda JCR, Posso YYA (2015). Factores de riesgo psicosociales en entidades prestadoras de servicios de
salud.

[8] Talavera-Velasco B, Luceño-Moreno L, Martín-García J, Navarro-Canedo A (2016). Factores de riesgo psicosocial en médicos de la provincia de
Valladolid: diferencias entre atención primaria y hospitalaria. Aten Primaria.

[9] Peñafiel MJM (2014). Servicio Social Obligatorio en Colombia: Incertidumbre de los recién
graduados en medicina. Rev Medica Risaralda.

[10] Colombia, Ministerio de la Protección Social, Pontificia Universidad Javeriana (2010). Batería de instrumentos para la evaluación de factores de riesgo
psicosocial.

[11] Colombia, Ministerio de Salud y Protección Social (2017). Resolución 2646 de 2008. Factores de riesgo psicosocial en el trabajo y
determinación del origen de las patologías causadas por el estrés
ocupacional.

[12] Flórez Pérez JC, Jiménez Palacio JC, Valencia Castañeda AJ, Garzón Duque MO (2020). Características psicosociales extralaborales e intralaborales de un grupo
de médicos en el servicio social obligatorio.

[13] Uribe Rodríguez AF, Martínez Rozo AM (2014). Factores psicosociales intralaborales en profesionales con personal a cargo
en la ciudad de Bucaramanga. Informes Psicol.

[14] Morillo Gómez AP, Morillo Gómez MM, Torres Urbano DC (2021). Riesgos psicosociales en el recurso humano del Hospital José María
Hernández (II Nivel) durante la emergencia sanitaria por Covid-19.

[15] López Góngora DC, Bohórquez Martínez F, Chitan Almeida ME (2021). Propuesta de plan de intervención para los factores de riesgos psicosocial
en médicos generales del servicio de urgencias y hospitalización expuestos
a la emergencia sanitaria COVID-19 en Los Cobos Medical Center de la ciudad de
Bogotá.

[16] Lozano AJP, Quitian GSG, Ríos JPD (2022). Factores de riesgo psicosocial presentes en el personal sanitario de primera
línea en atención de pacientes COVID-19 en Colombia año 2020 al
2021.

[17] Anaya LSG, García CP (2013). Riesgos psicosociales y sus efectos en el personal del sector salud en Colombia.
Un estudio comparativo.

[18] Ramírez MG, Barrera PAT (2022). Análisis de los factores de riesgo psicosocial presentados en trabajadores
de la salud en una Empresa Social del Estado del municipio de Sogamoso
Boyacá.

